# Not all synesthetes are alike: spatial vs. visual dimensions of sequence-space synesthesia

**DOI:** 10.3389/fpsyg.2014.01171

**Published:** 2014-10-30

**Authors:** Clare N. Jonas, Mark C. Price

**Affiliations:** ^1^School of Psychology, University of East LondonLondon, UK; ^2^Faculty of Psychology, University of BergenBergen, Norway

**Keywords:** synesthesia, spatial imagery, visual imagery, mental imagery, prevalence

## The variability of sequence-space synesthesia

Sequence-space synesthesia (SSS) is a common condition in which ordinal sequences such as months, numbers or the letters of the alphabet are perceived to occupy spatial locations in the mind's eye or peripersonal or extrapersonal space (e.g., Price and Mentzoni, 2008; Jonas and Jarick, 2013). For example, thinking about a month may elicit the visuospatial impression of a circular arrangement of the months, or hearing a numeral may elicit a specifically shaped number line. These “spatial forms” are typically thought to be consistent over time within an individual (e.g., Smilek et al., 2007), though they can actually evolve (Price and Pearson, 2013; Gould et al., 2014; Price, 2014; see also Simner, 2012; Meier et al., 2014). They are also idiosyncratic, with synesthetes reporting many different shapes of varying complexity (Galton, 1880; Phillips, 1897) that are experienced outside the body (i.e., projected) or in the mind's eye (i.e., associated; Dixon et al., 2004; Smilek et al., 2007; Ward et al., 2007).

SSS can vary along several dimensions, including the projector-associator distinction, automaticity, visual salience, and type of spatial transformation that can be applied to the spatial form (e.g., Price, 2013; Price and Mattingley, 2013). Since SSS could be considered a variety of visuospatial mental imagery, these individual differences may reflect known fractionation of imagery processes and skills (Price, 2013; Price and Pearson, 2013). However, a thorough and empirically grounded taxonomy for individual differences in SSS is missing (Price, 2014). We explore here a possible systematization for one area of these individual differences—the visual and spatial qualities of SSS. We further suggest this can help to classify synesthetes as experimental participants and perhaps explain some inconsistencies in published data.

## Classifying the visuospatial nature of sequence-space synesthesia

The visuospatial experiences of SSS are often referred to as *spatial* forms. For some synesthetes the forms are indeed a felt impression of spatial locations with minimal visual content. Others, however, report having a *visual* impression of their forms (Eagleman, 2009; Price, 2013); examples of detailed visual content such as texture, color, written text, and, associated visual images are often found in reports on SSS (e.g., Seron et al., 1992; Jonas et al., 2011; Gould et al., 2014; Price, 2014). It has been speculated (Price, 2013; Price and Pearson, 2013) that this variation reflects the distinction, from research on non-synesthetic imagery, between (1) *spatial imagery* of explicit spatial relationships that may be detailed and complex, which at its most sophisticated level takes the form of a spatial map with flexible viewpoints, (2) *visual imagery* that depicts visual appearance and more holistically represents visual surface properties. These complementary aspects of visuospatial imagery are tapped by different behavioral tests, are associated with separable working memory modules, and are implemented by separable neural networks, with spatial vs. visual components reflecting the dorsal vs. ventral streams of visual processing (Hegarty, 2004; Mazard et al., 2004; Kosslyn et al., 2007).

Here we suggest refining the visual vs. spatial imagery distinction as previously applied to SSS. It may be too simplistic to characterize synesthetes' forms as being *either* visual or spatial (as Price, 2014, suggested). Instead, we should characterize an individual's SSS along *both* visual and spatial dimensions. If these are orthogonal at the level of the individual, the synesthete may be independently high or low on each dimension.

Visual vs. spatial dimensions would respectively reflect an emphasis of ventral vs. dorsal stream activation in mediating synaesthetic imagery. This is consistent both with the view that SSS is continuous with normal visuospatial imagery (Price and Mattingley, 2013; Price and Pearson, 2013), and with proposals that SSS derives from functionally or structurally abnormal neural connectivity. Eagleman's (2009) suggestion that SSS is mediated by unusual connectivity to ventral stream representations could account for the visual aspects of SSS that he emphasizes. Suggestions that SSS derives from dorsal stream connectivity between spatial and magnitude representation in parietal cortex (e.g., Tang et al., 2008; Hubbard et al., 2011), could by contrast be more relevant for spatial aspects.

### Spatial dimension

This dimension characterizes the extent to which spatial forms can be construed as spatial models where people have explicit introspective access to the relative positions of sequence members. A synesthete low on this dimension has a form viewed consistently from the same vantage point, with low spatial resolution. A synesthete high on this dimension has a more explicit spatial model which will facilitate spatial transformations and allow forms to be seen from multiple viewpoints (Burgess, 2006), even if they have a typical viewpoint. The spatial representation may have vague sketch-like visual qualities but, as spatial sensation is created multi-modally, it could also occur without them.

One example of this distinction is seen in SSS for months, where some synesthetes report that their form is always located in the same space, while others report movement of the calendar or the self in relation to it over the year (Smilek et al., 2007). A more unusual example of spatial transformation comes from a synesthete (Jarick et al., 2009, 2010, 2013) who views her month form from different vantage points depending on whether she hears or reads the name of a month.

Different types of sequence may encourage different degrees of introspective access, along with different degrees and varieties of spatial transformation. For example, the first author (who has SSS) finds that examining spatial forms (e.g., her form for exam grades) improves introspective access. In terms of transformation, whereas SSS for months is often reported to move spontaneously with the passage of time, transformation of number forms (containing potentially infinite sequence members) typically involves more effortful “focus” or “zooming in” (Seron et al., 1992). By contrast, SSS for the alphabet (e.g., Jonas et al., 2011) may make low demands on spatial transformation because the alphabet does not change over time and is constrained enough to be seen from one viewpoint.

### Visual dimension

For synesthetes with low visual experience, spatial forms will seem to occupy space, but lack depictive visual quality. By contrast, synesthetes with high visual experience “see” their spatial form in visual detail from a particular viewpoint in their mind's eye, or in peripersonal or extrapersonal space (converging respectively with Ward et al. (2007), classification of grapheme-color synesthetes as see-associators, experiencing colors in their minds' eye, or projectors, whose graphemes have colors “out there” in the world).

### Orthogonal dimensions

The relation between these proposed spatial and visual dimensions of SSS experience remains to be empirically established. However, taking SSS for months as an example, we can envisage a 2 × 2 matrix containing the four extreme combinations of high and low spatial and visual characteristics (Table 1). Gradations between these extremes are also possible.

**Table 1:**
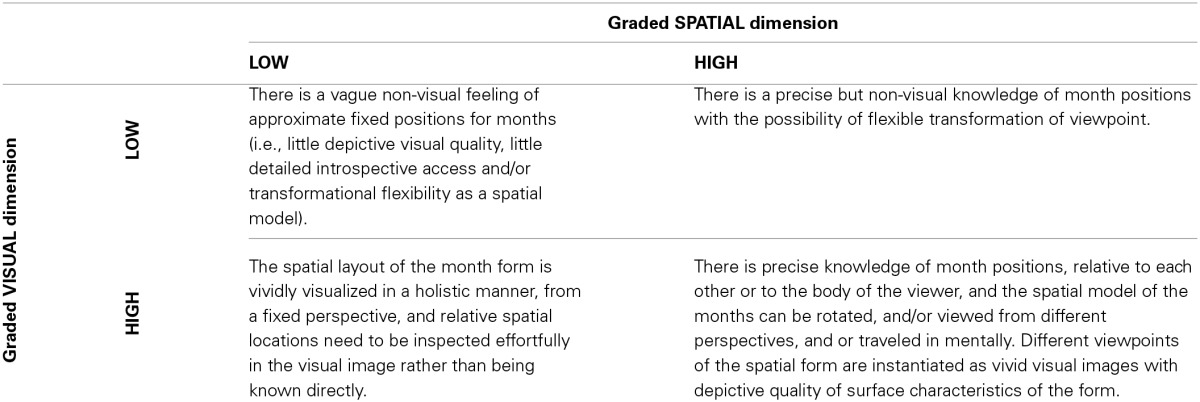
**Characterisation of SSS for calendar months that fall on low vs. high ends of orthogonal visual and spatial dimensions**.

## Using the visual/spatial classification to explain conflicting findings

### Prevalence of SSS

Given the assumption that SSS is consistent over at least short periods of time, a common method of verifying SSS is to quantify this consistency because, even with a very good memory, a non-synesthete would be unlikely to mimic the consistency arising from perceiving a spatial form.

The least stringent consistency test, used by Sagiv et al. (2006, *N* = 311), is to ask participants to draw their spatial associations twice, weeks or months apart, and to assess whether the drawings match. Sagiv et al. estimated prevalences of 20% for SSS involving time (days and/or months), 12% for SSS involving numbers, and 15% for SSS involving the alphabet (totaling 29% prevalence for any of the three types).

More stringent methods have since been developed, including Brang et al. (2010) within-session technique of asking participants with SSS for months to “project” their form onto a computer screen and indicate month location with mouse-clicks (each month was tested five times). This yielded a prevalence estimate of 2.2%, defined as the proportion of participants (*N* = 183) whose consistency was > 1.96 *SD* above the mean. This estimate is not directly comparable with Sagiv et al.'s estimate of 20% Brang et al. focused on months while Sagiv et al. made no distinction between days and months. Additionally, Brang et al.'s classification of participants as synesthetes if they showed outlier consistency is far more stringent than the criterion adopted by Sagiv et al. and as Brang et al. admitted, may have been overly conservative. Other studies have obtained estimates intermediate between these extremes (e.g., Chun and Hupé, 2013), but here we wish to raise the point that an additional cause of widely varying prevalence estimates may be that different methods selectively target different sub-types of spatial form.

For example, “projecting” one's month calendar onto a screen is likely easier for synesthetes high on the spatial dimension, who can view and rescale their calendar. Other synesthetes could be missed, and Brang et al. notably reported that some kind of mental layout for the months was verbally reported for 44% of their sample. Sagiv et al.'s drawing method may, by contrast, be easier for synesthetes high on the visual dimension. If highly visual SSS were more common than highly spatial SSS, then it is perhaps unsurprising that visually-based prevalence estimates such as that of Sagiv et al. are higher than spatially-based estimates such as that of Brang et al. It is indeed likely that visual SSS is most common: Strong experience of visual imagery seems more prevalent than strong spatial imagery in the general population (Chabris et al., 2006; Blazhenkova and Kozhevnikov, 2009), and several studies have found that samples of people with SSS—who were more neutrally recruited via verbal report—show elevated self-report scores for visual, but not spatial, imagery (Price, 2009; Rizza and Price, 2012; Meier and Rothen, 2013).

### Behavioral measures of mental imagery in people with SSS

Although high self-reported visual imagery scores among synesthetes seem replicable, results from behavioral tests of visuospatial imagery have been mixed (for further discussion see Simner, 2013; Price, 2013). Simner et al. (2009) found increased accuracy in synesthetes with time-related forms compared to controls on Benton's test of 3D praxis, VOSP progressive silhouettes, and 3D mental rotation. Brang et al. (2013) reported increased accuracy in time-space synesthetes performing 2D mental rotation of letters compared to non-synesthetes. However, Rizza and Price (2012) reported that time-space synesthetes performed no better than non-synesthetes on 3D imagery tests of paper folding and mental rotation.

To distinguish between rival explanations for these discrepancies (e.g., demand characteristics; lack of power; non-equivalence of tasks in different studies), further replication is needed with larger sample sizes and multiple behavioral imagery tests. However, we suggest that individual differences in synesthetes along spatial and visual dimensions could contribute to varied findings. For example, synaesthetes who are low on our visual dimension may not perform 3D mental rotation unusually well because this test correlates with self-reported spatial rather than visual imagery (e.g., Blazhenkova and Kozhevnikov, 2010). This may have been the case for the study by Rizza and Price (2012). If so-called “spatial” forms are often visual in nature (Eagleman, 2009; Price, 2013), and if such forms are more common than highly spatial variants, then it is plausible that small, randomly-selected SSS samples will contain few highly spatial participants. However, if the recruitment method favors spatial SSS, then a synesthete advantage for mental rotation may be obtained because such participants are by definition good at spatial transformation.

## Conclusion

We have suggested that characterizing SSS in terms of its orthogonal spatial vs. visual properties may capture some of the ways in which this experience varies between individuals. We have also speculated that failure to make this type of distinction between individual synesthetes may contribute to widely differing prevalence estimates and to divergent claims about the visuospatial skills associated with SSS. Further defining and refining empirical methods for classifying participants along spatial and visual dimensions would provide a helpful way to screen participants in future studies addressing the prevalence, behavioral correlates and neurocognitive basis of this condition.

### Conflict of interest statement

The authors declare that the research was conducted in the absence of any commercial or financial relationships that could be construed as a potential conflict of interest.
